# Gender Differences in Resistance to Schooling: The Role of Dynamic Peer-Influence and Selection Processes

**DOI:** 10.1007/s10964-017-0696-2

**Published:** 2017-05-30

**Authors:** Sara Geven, Jan O. Jonsson, Frank van Tubergen

**Affiliations:** 1 Department of Sociology, University of Amsterdam, Nieuwe Achtergracht 166, 1018 WV Amsterdam, The Netherlands; 2 University of Oxford, Nuffield College, New Road, Oxford, OX1 1NF UK; 3 Institute for Futures Studies, Box 591, 101 31 Stockholm, Sweden; 4 Department of Sociology, Utrecht University, Padualaan 14, 3584 CH Utrecht, The Netherlands; 5 Department of Sociology & Social Work, King Abdul Aziz University, Abdullah Suleiman Street, Al Jamiaa District, 80200 Saudi Arabia

**Keywords:** Gender gap, Student resistance, Peer influence, Dynamic social network analysis

## Abstract

Boys engage in notably higher levels of resistance to schooling than girls. While scholars argue that peer processes contribute to this gender gap, this claim has not been tested with longitudinal quantitative data. This study fills this lacuna by examining the role of dynamic peer-selection and influence processes in the gender gap in resistance to schooling (i.e., arguing with teachers, skipping class, not putting effort into school, receiving punishments at school, and coming late to class) with two-wave panel data. We expect that, compared to girls, boys are more exposed and more responsive to peers who exhibit resistant behavior. We estimate hybrid models on 5448 students from 251 school classes in Sweden (14–15 years, 49% boys), and stochastic actor-based models (SIENA) on a subsample of these data (2480 students in 98 classes; 49% boys). We find that boys are more exposed to resistant friends than girls, and that adolescents are influenced by the resistant behavior of friends. These peer processes do not contribute to a widening of the gender gap in resistance to schooling, yet they contribute somewhat to the persistence of the initial gender gap. Boys are not more responsive to the resistant behavior of friends than girls. Instead, girls are influenced more by the resistant behavior of lower status friends than boys. This explains to some extent why boys increase their resistance to schooling more over time. All in all, peer-influence and selection processes seem to play a minor role in gender differences in resistance to schooling. These findings nuance under investigated claims that have been made in the literature.

## Introduction

In highly developed countries, girls have been academically outperforming boys at least since the 1990s. Girls obtain higher reading and language test scores, get higher grade point averages, are less likely to drop out of school, and more often enter into higher education than boys (Buchmann et al. 2008). One possible factor underlying these differences is the tendency for boys to exhibit more resistance to schooling (Legewie and DiPrete 2012; Hadjar and Buchmann 2016). Student resistance to schooling refers to a lack of adherence to school rules and norms, such as defying teacher authority and refusing to put effort into school work (cf. McFarland 2001). This is sometimes also labelled behavioral disengagement and is generally related to lower school results and drop-out (Fredricks et al. 2004).

This study addresses the question of why boys are more resistant to schooling than girls. Previous research has highlighted the importance of peer processes for student resistance (McFarland 2001), as well as for the gender gap in it (Driessen and van Langen 2013). Qualitative studies suggest that, compared to girls, boys are more exposed to anti-school attitudes and behaviors in their peer groups and experience more pressure from their peers to exhibit anti-school attitudes and behaviors (e.g., Francis 1999; Warrington et al. 2000). Only a few quantitative studies have built on these research findings, and all of them are cross-sectional. These studies use compositional characteristics of classes or schools as a proxy for the extent to which boys and girls are exposed to peers with anti-school attitudes and behaviors. For example, boys’ and girls’ school attitudes and behavior have been compared across schools differing in their gender composition (Demanet et al. 2013; Van Houtte 2004) or average socio-economic status composition (Legewie and DiPrete 2012).

We improve on previous quantitative studies on the role of peers in gender differences in student resistance in two important ways. First, we study gender differences in peer selection and influence processes directly, instead of proxying them by compositional effects. Second, we use a longitudinal approach to examine the role of classroom peers on gender differences in school resistance. Peer processes are dynamic, warranting a longitudinal approach, which allows us to make better inferences about the causal direction of the relationships between the behavior of peers and that of adolescents (Hallinan 1981).

Adolescents may behave in similar ways as their classroom peers for three reasons. First, adolescents tend to adjust their behavior to the behavior of their classroom peers (i.e., peer influence effects). Second, adolescents behave in similar ways as their friends in class, since adolescents are inclined to befriend peers who are similar to them, and to unfriend peers who are dissimilar to them (i.e., peer selection and deselection effects). Third, adolescents may be similar to their peers for other reasons than peer processes. Students who attend the same school or class tend to be exposed to similar contexts (e.g., neighborhoods) or come from similar backgrounds (i.e., contextual or background effects), which causes them to exhibit similar behaviors. It is impossible to disentangle these different effects with cross-sectional data. We will apply advanced longitudinal statistical techniques, including longitudinal social network analysis (i.e., Simulation Investigation for Empirical Network Analysis, SIENA), to analyze large scale panel data on adolescents’ friendships in class and their school behavior in Sweden, drawn from the CILS4EU project (Kalter et al. 2013).

## Two Peer Effect Explanations

The gender gap in school resistance may be explained by a differential peer exposure or a differential peer reaction mechanism (Haynie et al. 2014). The exposure explanation implies that girls are surrounded, and thus influenced, by less deviant peers than boys. According to the peer reaction explanation, girls are less susceptible to the influence of deviant peer norms than boys, which causes boys to be more deviant than girls.

In this study, “peers” refer to befriended and non-befriended classmates. We focus on classmates, since student resistance is often enacted in class and classmates play a pivotal role in a student’s decision to engage in resistant behavior (McFarland 2001). We examine the role of befriended and non-befriended classmates separately, since we assume that both peer groups could be influential, yet for different reasons. According to normative social influence theory, people are influenced by the behavior of peers to avoid social sanctions and to gain social approval by them (Cialdini and Goldstein 2004). Friends in class could be influential because they are valued peers, and adolescents strive to maintain their friendships (Hallinan 1981). Non-befriended classmates could be influential because these classmates can still become friends and adolescents may try to impress these potential friends (Frank et al. 2008); or because adolescents try to avoid social sanctions in class, such as mockery. Compared to friends, non-friends may be less accepting of “inappropriate” behavior (Müller et al. 2016). Finally, the behavior of non-befriended classmates may set a norm to which students want to conform.

In previous studies, it is unclear whether befriended or non-befriended peers are more influential with respect to boys’ and girls’ school outcomes. For example, Molloy et al. (2011) suggest that adolescents’ effort in school is influenced more by friends in class than by classmates that adolescents are less strongly connected to. However, a study by Frank et al. (2008) indicates that girls’ decision to advance in math is not influenced by friends in class, but only by more distant peer groups (i.e., female schoolmates and female students who follow the same courses). Finally, Müller et al. (2016) find that the perceived disruptive behavior of all classmates, high-status classmates, and friends in class equally influence a student’s own disruptive behavior. By studying befriended and non-befriended classmates separately—rather than assuming that they are equally influential—we aim to gain a deeper understanding of the role of peers in the gender gap in student resistance to schooling.

### Differential Peer Exposure Explanation

While boys and girls who attend the same class are exposed to the same classmates, they tend to befriend and interact with different classmates. Boys may not deliberately befriend peers who exhibit higher levels of resistant behavior, yet other friendship selection processes are expected to expose boys to higher levels of student resistance in their friendship group than girls. First, gender homophily—the tendency for boys to befriend boys, and for girls to befriend girls (McPherson et al. 2001)—could lead to gender differences in the exposure to resistant friends, simply because boys generally exhibit more resistant behavior than girls (Buchmann et al. 2008). Second, we expect that adolescents befriend others with similar values and tastes (McPherson et al. 2001), including resistant behavior. Previous studies have found such tendencies with respect to homework behavior and attentive behavior in school (Geven et al. 2013), externalizing problem behavior in school (Fortuin et al. 2015), truancy (Rambaran et al. 2016), and academic achievement (Flashman 2012; Gremmen et al. 2017). Classmates’ resistance to schooling is highly visible in class, and adolescents may use this behavior as a signal of similarity. Moreover, adolescents might engage in resistant behavior, such as skipping class, together with other classmates. Such shared “activities” can lead to friendships. Since boys generally show more resistance to schooling than girls, homophily with respect to school resistance implies that boys—more often than girls—will befriend peers who exhibit higher levels of school resistance.

**Fig. 1:**
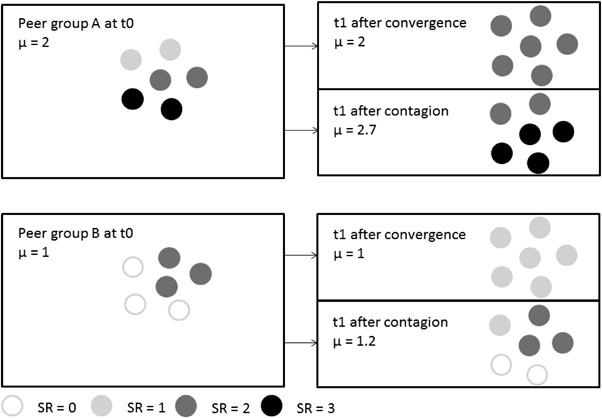
Convergence and contagion processes in two peer groups. *Note*: Each circle represents a person. The color of the circle indicates a person’s level of student resistance (SR). Darker colors imply higher levels of student resistance. Group A represents a male friendship group, and group B a female friendship group. The right pictures show the level of SR after convergence (*top picture*) and after contagion (*bottom picture*) at time point 1. In male friendship group the initial level of SR is higher than in the female friendship group. After convergence, the average SR remains the same in both groups, and the gender difference in SR is not altered. After contagion, people in group A increase their SR more than people in group B. In other words: the gender gap increases. (Color online)

There are two ways in which gender differences in the exposure to resistant friends could contribute to the gender gap in student resistance. First, they could lead to an increase in the gender gap in resistance to schooling over time. This may occur if adolescents who are exposed to more resistant friends are inclined to increase their resistance to schooling more. This type of influence processes is also referred to as contagion (see Fig. 1). Engagement in minor forms of deviant behavior is related to social rewards and status in adolescent peer groups, as it is a way to show autonomy from adults (Moffitt 1993). Resistance to schooling can be seen as a form of deviance that is related to status in some adolescent friendship groups (Demanet and Van Houtte 2012). In friendship groups in which the level of resistant behavior is higher, adolescents may experience more stimulation or pressure to increase their own resistance to schooling, learning from their friends that this is a way to gain status. Since boys are generally embedded in friendship groups in which the level of resistant behavior is higher, boys will increase their resistance to schooling more than girls. Consequently, the gender gap in resistance to schooling will increase.

Second, gender differences in the exposure to friends could lead to the persistence of initial gender differences in resistance to schooling. This may occur if adolescents tend to grow similar to the average behavior of their friends. More specifically, adolescents will decrease their resistant behavior when they are exposed to friends who exhibit less resistant behavior than they do, while they will increase their resistant behavior when they are exposed to friends who exhibit more resistant behavior than they do (see Fig. 1). This type of influence process is also referred to as convergence. Research has found support for convergence processes among friends with respect to attention in class, doing homework (Geven et al. 2013), truancy (Rambaran et al. 2016), and externalizing problem behavior (Fortuin et al. 2015). When girls grow similar to the average behavior of their primarily female friends, and boys grow similar to the average behavior of their primarily male friends, the gender gap in resistance to schooling will remain stable over time (see Fig. 1). In line with this, simulation studies indicate that, when people interact with others with similar opinions (homophily) and their own opinions converge to those of their interaction partners, segregated opinion clusters emerge (Mäs and Flache 2013). However, the difference in the average opinions of these clusters do not become larger over time.

### Differential Peer Reaction Explanation

Normative social influence theory predicts that the influence of befriended and non-befriended classmates is stronger with respect to types of behavior that lead to social rewards. In general, engaging in gender-typical behavior is more socially accepted than engaging in gender atypical behavior (Rose et al. 2011). According to Adler et al. (1992), boys’ gender roles have traditionally been marked by a more “active” nature than that of girls. Ethnographic work indicates that pro-school behavior is in conflict with the male image (e.g., Francis 1999), and that boys receive their status from, among other things, breaking the rules and disobeying adult authority such as teachers (e.g. Jackson 2003). Although performing well in school is neither for adolescent boys, nor for adolescent girls, an important source of social status, pro-school behaviors may be detrimental to boys’ status (Willis 1977). Compared to girls, boys risk social punishments by peers when they engage in pro-school behaviors and they experience more peer pressure to exhibit anti-school behaviors (Warrington et al. 2000).

Quantitative research has shown little support for gender differences in the relationship between academic performance or intelligence and social status (Rose et al. 2011). However, boys may not derive their social status from their low school performance, but rather from their active disengagement and rejection of school. In line with this, research shows that German adolescents allocate higher social status ratings to descriptions of male student showing low school effort than to descriptions of female students showing low school effort (Rentzsch et al. 2011). A recent German study finds no support for this relation, but shows that adolescents associate descriptions of students showing low school effort with masculinity and boy-typicality (Heyder and Kessels 2016). This may imply that showing low school effort is more important for boys than for girls. All in all, we assume that exhibiting resistant behavior carries more social value for boys than for girls, and it is also likely that boys will be influenced more by the resistant behavior of their befriended and non-befriended classmates.

However, social influence theory suggests that boys may not be more responsive to the resistant behavior of all their peers, but primarily to high status peers because the imitation of their behavior is related to greater social rewards (Shi and Xie 2012). Students may believe that the emulation of high status peers enhances their own social status (Cohen and Prinstein 2006) or that it increases their likelihood for inclusion in (a) high-status peer group(s) (Dijkstra et al. 2008). Moreover, imitating high status peers can lead to feelings of reflected glory (i.e., feelings of success related to the association with successful others) (Cialdini et al. 1976; Dijkstra et al. 2008). Because the engagement in resistant behavior seems more related to the social status of boys than to that of girls, we expect that boys will be influenced more by the resistant behavior of higher status peers than girls.

It is also possible that boys and girls generally respond to the resistant behavior of different classmates. According to social influence theory, people are influenced more by those who are similar to themselves with respect to central aspects of their identity (e.g., sex). The behavior of in-group members forms a reference on how one should behave as a member of the group (Mason et al. 2007). For example, boys look at other boys to learn how to behave as males. In-group members are particularly influential on attitudes and behaviors that are important to the identity of the group (Wood 2000). Since adolescents may express their femininity or masculinity through their school behavior (Francis 1999), we expect that the school behavior of same-sex classmates has a stronger positive influence on adolescents’ school behavior than that of opposite-sex classmates.

The tendency to be primarily influenced by same-sex classmates could lead to an increasing or persistent gender gap in student resistance. If students are influenced by the resistant behavior of same-sex classmates via contagion, a feedback process may emerge that causes boys—who have a higher initial propensity to exhibit resistant behavior—to increase such behavior even more, leading to growing gender differences in student resistance. If adolescents are influenced to converge their resistant behavior to those of same-sex classmates, boys will adjust their behavior to the average norm for boys, while girls will adjust their behavior to the average norm for girls. This may lead to a stable gender gap in student resistance.

## Current Study

This study aims to examine the role of peer-influence and selection processes in school classes for gender differences in student resistance. We contribute to previous research by explicitly and directly assessing the role of befriended and non-befriended classmates, and by using longitudinal data and novel statistical methods that enable us to better identify influence effects.

We argue that gender differences in the exposure and the response to resistant classmates could lead to time-stable or even increasing gender differences in student resistance to schooling. More specifically, we hypothesize that the friends of boys exhibit higher levels of resistant behavior than the friends of girls (hypothesis 1a). This in turn will cause boys to exhibit higher levels of resistance to schooling than girls, since the resistant behavior of friends positively influences the resistant behavior of the adolescent (hypothesis 1b).

In addition, we expect that, compared to girls, boys are more positively influenced by the resistant behavior of their befriended (hypothesis 2a) and non-befriended classmates (hypothesis 2b), and that this is accentuated when these have a higher social status (hypothesis 3a and 3b). Finally, we hypothesize that adolescents are influenced more by the resistant behavior of same-sex classmates than that of opposite-sex classmates (hypothesis 4). We can only test this hypothesis for non-befriended classmates, as most adolescents do not have opposite-sex friends (more than 85% of the friendships in our data are same-sex friendships).

We test the hypotheses on two-wave panel data of Swedish adolescents (14–15 years old in wave 1). We believe that it may be possible to generalize the case of Sweden to other countries, as we expect that social influence and homophily are predominantly generic processes. Although gender equality is generally high in Sweden, a substantial gender segregation is apparent in educational choices, and girls have dominated higher education since the early 1980s (Jonsson 1999).

We conduct two different types of analyses to model student resistance to schooling… First, we perform hybrid models. Second, we use SIENA (Simulation Investigation for Empirical Network Analysis), a longitudinal social network approach, to retest all the hypotheses with respect to befriended classmates (hypothesis 1, 2a, and 3a). In the SIENA models we also model friendship selection processes in class as a dependent variable.

## Methods

### Participants

Data on Swedish adolescents are drawn from the CILS4EU data (Kalter et al. 2013). All participants attended 8th and 9th grade (wave 2) of comprehensive school, and a large majority of them went to a public school (Jonsson and Mood 2008). The data contain student reports on their friendships in class (i.e., complete network data) and their resistance to schooling.

CILS4EU used a multi-stage stratified sampling design. First, schools were divided into strata according to the proportion of minority students, oversampling immigrant-dense schools. Within strata, schools were randomly selected with the sampling probability being proportional to the number of students. Subsequently, all students in two randomly selected classes were invited to participate. Questionnaires were filled out in class and supervised by a professional interviewer.

In Sweden, 5025 students in 251 classes in 129 schools participated in the first wave in the school year of 2010–2011. The response rate was 76.8% at the school-level and 86.1% at the student-level. All but one school (98.5%) participated again 1 year later. 4110 students participated in both waves, and 5448 in one of the waves.

The Swedish data are highly suitable for a longitudinal investigation of peer processes in class. First, mobility across school classes is relatively low. On average 77% of a Swedish student’s classmates in the first wave were also his/her classmates in the subsequent wave. Second, the school class is a natural unit to which most educational activities are confined—students usually only mix with those from other classes in a few subjects. Relatedly, the class is an important context for friendship formation in school in Sweden. In the first wave of the data, 75% of all school-based friendships are friendships with classmates. Third, student participation rates are relatively high in both waves. Unfortunately, information on students in the Netherlands, Germany, and England in the CILS4EU data are much less appropriate for analyzing peer processes in class longitudinally (see Appendix A1), hence the focus on Sweden.

### Variables

#### Student resistance

Five items measure student resistance to schooling: the extent to which adolescents argue with teachers, get a punishment in school, skip class, come late to school, and put a lot of effort into school. Response categories are on a 5-point scale, and range from “never” to “every day” for the first four items, and from “strongly agree” to “strongly disagree” for the last item. Higher values thus imply higher student resistance. The five items load on one factor (Cronbach’s alpha 0.74 in wave 1 and 0.70 in wave 2), and item loadings are all above 0.6. For the hybrid model, student resistant behavior is measured by a student’s average score on the items. In SIENA only ordinal behavioral outcome variables with a maximum of ten categories can be analyzed. Hence, we round the average resistant behavior score to the nearest half, and recode this value to an ordinal scale that ranges from 1 to 9 (e.g., a score of 0 becomes 1, 0.5 becomes 2 etc.). The resulting ordinal variable correlates highly with the non-rounded variable (0.98 in both waves).

#### Predictors of student resistance

##### Boy

In SIENA, a positive effect indicates that, compared to girls, boys have a higher tendency to increase their resistance to schooling. In the hybrid model we estimate a time-constant and time-varying effect of gender, the latter by including an interaction between gender and time.

##### Resistance friends

Adolescents were asked to list their best friends in class (maximum of 5). The resistant behavior of friends is measured by friends’ average score on the student resistance variable. In SIENA we specify the influence of the behavior of friends with the “Average Alter” effect: If it is positive, an adolescent tends to increase his/her resistant behavior more when the average resistant behavior of his/her friends is higher. The average alter effect represents a “contagion” type of influence (Veenstra et al. 2013) (see Fig. 1).^1^

##### Resistance non-friends

In the hybrid models we estimate the effect of the resistant behavior of classmates who are not nominated as friends, using their average score on the student resistance scale.

##### Resistance male non-friends and resistance female nonfriends

In the final hybrid model we distinguish between the average student resistance of non-befriended males and that of non-befriended females.

##### Status friends and status non-friends

The higher number of incoming friendship ties (i.e., indegree), the higher social status. In SIENA we include the average indegree of the adolescent’s friends (i.e., the Popularity Alter effect). In the hybrid model we include the average indegree of friends (social status friends) and non-friends in class (social status non-friends).

#### Student resistance control variables

To correctly estimate social influence processes in SIENA, we control for the linear shape and the quadratic shape effects (Ripley et al. 2017). A positive linear shape effect implies that people are inclined to have high values on the dependent variable. A positive quadratic shape effect implies that students reinforce their own resistant behavior, whereas a negative quadratic shape effect implies that students self-correct their resistant behavior. In the hybrid models we include a time dummy to model whether students increase or decrease their resistance to schooling across the two waves.

We control for parental education. When parents value education and stimulate school work, adolescents might be influenced less by their friends’ resistance to schooling. Parental education is used as a proxy for parental values and support and is measured by the educational level of the parent with the highest acquired qualification. For most parents this information is obtained from register data from Statistics Sweden.

Finally, in the hybrid models, we include the proportion of non-befriended classmates that are boys (Proportion non-befriended boys) and the social status of the adolescent as measured by his/her indegree. In the SIENA models we try to not include too many (unnecessary) control variables, as the statistical power of the analyses is somewhat limited. However, to make sure that we do not fail to include important controls, we score-type tested two possible additional control variables in SIENA (Ripley et al. 2017), namely the adolescent’s indegree and his/her number of outgoing friendship nominations (i.e., outdegree). None of these were significant predictors of student resistance to schooling (results available upon request).

#### Friendships networks

Student friendship nominations in class (up to 5) are modelled as a dependent variable in the SIENA model.

#### Predictors of friendship networks

##### Sex homophily

This effect indicates whether adolescents are more likely to befriend same-sex classmates than opposite-sex classmates.

##### Resistance homophily

This effect indicates the tendency to befriend classmates who exhibit similar levels of resistance to schooling.

#### Friendship network control variables

Homophily effects are dependent on characteristics of the adolescent (i.e., ego) and his/her friend (i.e., alter). Hence, we include the effect of adolescents’ resistant behavior on their tendency to nominate friends (Resistance ego) and to be nominated as a friend (Resistance alter). Similarly, we control for the effect of a student’s sex on the tendency to nominate friends (Boy ego) and to be nominated as a friend (Boy alter).

Student national-origin background might affect friendship selection processes. We estimate whether respondents who have two Swedish-born parents (rather than one or two foreign-born parents) are more likely to nominate classmates as a friend (Native Ego) and to be nominated as a friend (Native Alter). The National-Origin homophily effect indicates whether adolescents tend to befriend classmates of the same national-origin background, as defined by the country of birth of the respondent’s parents. When parents were not born in the same country, the background of the student is based on the foreign-born parent. When parents were born in different foreign countries, the background of the student is based on the mother’s country of birth. The national-origin background of the respondent is based on the respondent’s country of birth when the parental country of birth is missing (<2 %).

We include several structural network effects. Not accounting for these effects may lead to biased estimates (Ripley et al. 2017). The outdegree effect expresses a student’s tendency to nominate classmates as a friend (Steglich et al. 2010). Reciprocity refers to the inclination to reciprocate friendship ties; transitive triplets accounts for the fact that people tend to befriend friends of friends, and the 3-cycle effect controls for egalitarian triadic closure (which means that all members in a triad—three actors—are connected, and receive an equal number of nominations). Finally, we include two additional outdegree effects, as initial Goodness of Fit tests indicated that the SIENA models underestimated the number of students with low outdegrees. We include the outdegree activity and the outdegree activity sqrt effects. These are, respectively, the squared outdegree of the actor, and the outdegree^1.5, representing non-linear preferences in the number of outgoing friendship ties.

### Plan of Analyses

#### Hybrid models

We estimate hybrid models in Stata 14. Hybrid models combine the advantages of fixed effect and random effect models (Allison 2009). Similar to fixed effect models, they allow for the estimation of changes within individuals, while accounting for time-invariant effects of time-invariant characteristics of people (i.e., contextual or background effects, such as family or neighborhood characteristics). This is important as these may cause (some of) the similarity between adolescents and their classmates.

Similar to random effect models, hybrid models allow for the estimation of time-invariant effects of time-invariant variables. Hence, we are able to estimate the extent to which boys generally exhibit higher levels of resistant behavior than girls (i.e., the initial gender gap) and to explore the extent to which peer processes contribute to the stability of the initial gender gap.

We account for the nested structure of the data (i.e., time points are nested in students, who are nested in classes). A hybrid model with this nested structure can be expressed by the following formula:

$$\begin{array}{ccccc}\\ {y_{tic}}{\rm{ = }} & {\beta _{00}} + {\beta _{01}}{x_{1tic}} + {\beta _{10}}{z_{1ic}} + {\beta _{02}}\left( {{x_{2tic}} - {{\bar x}_{2ic}}} \right)\\ & + {\beta _{20}}{x_{2ic}} + {\beta _{11}}{x_{1tic}}{Z_{1ic}} + {v_{0c}} + {u_{0ic}} + {e_{tic}}\\ \end{array}$$

In this formula *y*_*tic*_ refers to the resistant behavior at time point *t* of student *i* in class *c*. *β*_10_ is the estimate of an effect of a time-varying variable, such as time. *β*_10_ is the effect of a time-constant variable, such as gender. *β*_11_ represents the effect of an interaction between time and gender (i.e., the extent to which boys increase their resistant behavior more than girls over time). *β*_02_ represents the within-individual effect of the time varying variable *x*_2_, such as the resistant behavior of befriended classmates, while *β*_20_ is the between-individual effect of variable *x*_2._ More specifically, *β*_20_ is the effect of the respondent’s mean on the time-varying characteristic; *β*_02_ is the effect of respondent’s deviation from his/her personal mean at a specific time point.

We estimate hybrid models with robust standard errors (i.e., Huber White estimator), to correct for non-normally distributed residual errors. We analyze data of all students who participated in at least one of the two waves. We impute missing values by means of multiple imputation with chained equations. We impute ten datasets in a wide format, so that a non-missing value on a variable in one wave can be used to impute a missing value on that same variable in another wave (Young and Johnson 2015). The imputation model includes all independent variables, the dependent variable, and a dummy for the student’s school class.

#### SIENA models

In the hybrid models we are not able to appropriately disentangle the influence of friends from friendship selection processes. Hence, we retest the hypotheses with respect to befriended classmates in SIENA, specifically designed to separate these processes by using longitudinal information and stochastic actor-oriented modeling (Steglich et al. 2010). SIENA has a “friendship selection part” in which changes in student friendship networks are modelled as a dependent variable; and a “behavioral part” in which changes in student resistance are modeled as a dependent variable. The evolution of student networks and student behavior are treated as endogenous and interdependent processes. SIENA assumes that changes in people’s behavior and their network may occur in between observation points (Steglich et al. 2010). More specifically, it simulates the changes in the network and the behavior of respondents in between the waves. Estimates in SIENA are based on these simulations. It is possible to control for other important endogenous network processes, such as the inclination to reciprocate friendships and to befriend friends of friends, that may lead to behavioral similarity among friends.

There are also disadvantages of SIENA in comparison to the hybrid models. First, it is not possible to account for the effect of unobserved time-invariant characteristics in SIENA (Steglich et al. 2010). Second, we cannot model the influence of the behavior of non-befriended classmates.^2^ Finally, the SIENA data requirements are rather stringent. This means that in many studies, including the present one, researchers can only rely on a subsample of their data.

SIENA uses data on relationships between people within a certain setting, such as a school class (i.e., complete network data). It requires that no more than 40% of the students join or leave the class after the first wave (Lubbers et al. 2011) and that at least 80% of the students participate in each wave (Ripley et al. 2017). Moreover, for estimates to be reliable, friendship networks have to be stable enough (as indicated by a Jaccard index >0.2) (Snijders et al. 2010).^3^ Two thousand six hundred and seventy one adolescents in 108 classes and 78 schools meet these data requirements (46% of the total sample). In addition, we drop 10 classes (191 students), because they cause convergence problems.^4^ In Appendix A2 we provide information on the extent to which the students that are included in the SIENA models differ from the students that are excluded. Although several students are excluded, the SIENA sample is unique in its size and representativeness. Most previous studies that use social network techniques rely on samples from far smaller and more restricted datasets, e.g., all students from a couple of schools (e.g., Haynie et al. 2014) or students from classes in a particular city (e.g., Rambaran et al. 2013).

The CILS4EU data contain multiple networks (i.e., school classes). Ideally, these should be analyzed separately, and subsequently be combined in a meta-analysis (Snijders and Baerveldt 2003). However, we do not have enough statistical power to apply this approach, as the average school class only consists of 25 students, and we only have two waves of data.^5^ Hence, we take a two-step approach (see Fortuin et al. 2015). First, we combine classes together in multiple multi-group analyses in SIENA. Second, we perform a meta-analyses on these multi-group analyses.

Classes that are grouped together with the multi-group approach in SIENA are not assumed to be related to each other; ties across the classes are not permitted. However, all parameters, except for the rate parameter, are assumed to be the same for classes that are combined (see Appendix A3). Hence, we combine classes in a multi-group model on the basis of their gender composition, as the gender composition may impact the parameters of the hypothesized effects (i.e., gender homophily in friendships, student resistance homophily in friendships, gender differences in resistant behavior, and (gendered) influence processes with respect to resistant behavior among friends) and the general level of resistant behavior in class (Demanet et al. 2013). We sort the classes by their share of boys, and split the data in 18 groups of six classes (i.e., groups of about 150 students). We combine six classes in one multi-group model to ensure that we have enough statistical power. Because students were only allowed to nominate up to five classmates, we set the maximum outdegree for the simulated networks to five in the analyses.

We combine the 18 multi-group analyses in a meta-analyses. The meta-analyses provide a joint significance test and an estimate for each effect based on Snijders and Baerveldt’s (2003) method. The meta-analyses also indicate whether effects significantly vary across the 18 groups, i.e., across classes that differ in their gender composition. Finally, they provide Fisher-type tests that indicate whether a parameter is significantly smaller or larger than zero in any of the subgroups.

#### Testing the hypotheses

We are interested in the extent to which peer processes contribute to (time-stable and/or increasing) gender differences in resistance to schooling. Ideally, we want to test whether these gender differences are mediated by peer processes. Unfortunately, there are no formal mediation tests available in SIENA and multilevel mediation models on our data do not converge in Stata (i.e., note that we have a complex multi-level model with three-levels, various interactions, and multiple imputed data). Hence, we follow the approach by Stark (2015) who tests for mediation in SIENA by comparing estimates from a model with and a model without the hypothesized mediator(s). If the coefficient is reduced after the possible mediator(s) are included, there is support for mediation. In a first model, we examine gender differences in resistance to schooling. In a second, we test whether resistance to schooling is related to friends’ resistance (hypothesis 1b), and whether gender differences are reduced when accounting for friends’ resistance (hypothesis 1). In the hybrid models, we are able to examine the reduction in the time-stable gender difference, and the reduction in the increase in the gender difference over time. In the SIENA models, these two effects cannot be separated, and we examine whether the effect of gender on students’ likelihood to increase their resistance to schooling turns to insignificance. SIENA estimates are based on simulations and hence are slightly different in different models. Moreover, SIENA estimates are expressed as log-odds, which cannot be compared across models (Mood 2010). The gender estimate in the SIENA model may go up after the resistant behavior of friends is added to the model, because the unobserved heterogeneity in the model decreases.

We also test whether boys are more exposed to resistant friends than girls (hypothesis 1a). We perform a t-test to examine gender differences in the resistant behavior of friends. Moreover, in the friendship selection part in SIENA, we test for friendship selection processes that are expected to be responsible for boys’ greater exposure to resistant friends. More specifically, we model the tendency of adolescents to befriend same-sex classmates and to befriend classmates who exhibit similar levels of resistance to schooling.

To test hypothesis 2a and 2b, we include an interaction between gender and the within-individual resistant behavior of (non-)friends in the hybrid model. Positive interaction effects indicate that an increase in the resistant behavior of (non-)friends is more positively related to an increase in boys’ resistance to schooling than that of girls. In SIENA, we test an interaction between the respondent’s gender and the resistance of friends by means of score-type tests (i.e., an interaction between the Average Alter effect and the Boy ego effect in the behavioral part of the SIENA model). A left-sided test indicates whether the effect is smaller than zero, and a right-sided test indicates whether the effect is larger than zero. Because the effect is tested twice, we use a significance level of α/2 (=0.05) (Ripley et al. 2017). Score-type tests do not provide an estimate for the interaction effect, but are preferred over directly estimating the effect, since the latter is likely to lead to convergence problems (Mercken et al. 2010).

In a subsequent model we test whether boys, as compared to girls, are more influenced by the resistant behavior of (non-)friends with a higher social status (hypothesis 3a and 3b). In the hybrid model, we include a three-way interaction between gender, the within-individual effect of the resistant behavior of (non-)friends, and the between-individual effect of the social status of (non-)friends.^6^ We control for all the two-way interaction effects that are underlying this three-way interaction. In SIENA, we use score-type tests for the three-way interaction between the respondent’s gender (i.e., Boy ego effect), the social status of friends (i.e., Popularity Alter effect), and the resistance of friends (i.e., Average Alter effect) as well as for all underlying two-way interactions.

In the final hybrid model, we test hypothesis 4. We include separate variables for the resistant behavior of non-befriended girls and boys. Moreover, we include interactions between the respondent’s gender and the within-individual effect of the resistant behavior of non-befriended boys and girls. We test whether an increase in the resistant behavior of non-befriended classmates of the same sex is more positively related to an increase in resistant behavior than the corresponding increase of those of the opposite sex.

## Results

### Descriptive Results

Table 1 presents the descriptives of the sample for the hybrid models (i.e., the full sample), and Table 2 shows the descriptives of the SIENA subsample. As expected, boys seem to exhibit higher levels of resistance to schooling than girls. In the full sample, boys’ level of resistance is 0.11 higher in wave 1, and 0.14 higher in wave 2. T-tests indicate that these gender differences are significant (wave 1: t(5011) = −6.676, *p* < 0.001; wave 2: t(4502) = −7.481, *p* < .001), and that boys increase their resistant behavior slightly more than girls over time (gender difference in increase is 0.034, t(4091) = −2.231, *p* = 0.026). Table 1 also indicates that the friends of boys exhibit higher levels of resistant behavior than the friends of girls. In line with hypothesis 1a, the resistant behavior of the friends of boys is 0.08 higher than the resistant behavior of the friends of girls in both waves (Table 1). T-tests indicate that these differences are significant (wave 1:t(4767) = −6.085, *p* *<* 0.001; wave 2:t(4383) = −5.912, *p* < .001).

**Table 1:** Descriptive statistics for the hybrid analyses. *N* individuals = 5448; *N* school classes = 251

	W1			W2		
	Mean (s.d.)	Range	% missing	Mean (s.d.)	Range	% missing
Time-varying variables						
Student resistance	0.72 (0.60)	0–4.0	7.98	0.75 (0.60)	0–4.0	16.94
Student resistance boys	0.78 (0.63)	0–4.0	7.90	0.82 (0.62)	0–4.0	16.14
Student resistance girls	0.67 (0.58)	0–3.2	7.36	0.68 (0.57)	0–4.0	17.86
Resistance friends	0.66 (0.44)	0–4	12.46	0.63 (0.45)	0–3.8	19.16
Resistance friends of boys	0.69 (0.44)	0–4	11.63	0.67 (0.46)	0–3.8	19.42
Resistance friends of girls	0.62 (0.43)	0–2.6	12.61	0.59 (0.43)	0–2.55	18.99
Resistance non-friends	0.73 (0.22)	0.1–2.3	12.46	0.75 (0.21)	0.26–1.56	19.16
Resistance male non-friends	0.79 (0.34)	0–3.2	12.67	0.82 (0.31)	0–2.6	19.95
Resistance female non-friends	0.67 (0.29)	0–2.1	12.74	0.70 (0.30)	0–2.2	19.97
Status friends	3.87 (1.63)	0–9.2	12.00	3.36 (1.74)	0–8.7	19.05
Status non-friends	2.52 (0.65)	0.4–4.7	12.00	2.42 (0.64)	0–4.5	19.05
Status adolescent	3.15 (1.95)	0–13	0	2.73 (1.89)	0–11	0
Proportion non-befriended boys	0.49 (0.14)	0.0–1.0	0	0.49 (0.14)	0.1–0.9	0
Time-constant variables						
Boy	.49	0/1	0.39			
Parental education	3.18 (1.47)	0–6	0.6

**Table 2:** Descriptive statistics for the SIENA analyses. *N* individuals = 2480; *N* school classes = 98

	W1			W2		
	Mean (s.d.)	Range	% missing	Mean (s.d.)	Range	% missing
Friendships						
Outdegree	3.811 (1.330)	0–5	11.45% absent 5.40 % join class in w2		3.554 (1.663)	11.65% absent 4.84% leave class in w2
Density	15.03% (3.079)	0–100		14.272% (3.171)	0–100	
Reciprocity	69.030% (10.031)	0–100		66.202% (10.351)	0–100	
Transitivity	55.717% (11.966)	0–100		54.780% (9.613)	0–100	
Proportion same-sex	90.004% (29.429)	0–100		85.878% (22.129)	0–100	
Resistance friends^a^	2.308 (0.827)	1–8		2.403 (0.775)	1–6	
Jaccard index (w1–w2)	0.445 (0.101)	0.220–0.823				
Student resistance adolescent	2.38 (1.22)	1–9	9.93%^b^	2.44 (1.23)	1–9	10.97%^b^
Student resistance boys	2.43 (1.19)	1–9	6.85%^b^	2.59 (1.28)	1–9	8.26%^b^
Student resistance girls	2.25 (1.24)	1–7	5.68%^b^	2.27 (1.154)	1–7	8.73%^b^
Individual independent variables^c^						
Boy	49.17%	1/0	4.96%			
Native	62.47%	1/0	4.48%			
Parental education	3.413 (1.432)	0–6	5.12%			

^a^ Given that a friends is nominated

^b^ Missingness is mainly due to the fact that people were absent. For the descriptives of wave 1 we excluded people who joined the school class in wave 2. For the descriptives of wave 2 we excluded people who left the class after wave 1

^c^ All individual-independent variables are time-invariant

### Hybrid Models

The results of the hybrid models are presented in Table 3. In the first model we include the effect of time, gender, the interaction between them, and the control variables. The model shows that boys’ level of resistance is 0.125 (0.21 of a standard deviation) higher than that of girls, and that boys increase their resistant behavior by 0.029 more than girls (i.e., boy*time interaction) between the two waves.

**Table 3:** Hybrid models on student resistance to schooling. *N* observations = 10,896; *N* individuals = 5448; *N* school classes = 251

	Model 1		Model 2		Model 3		Model 4		Model 5		Model 6		Model 7	
	Coef.	S.E.	Coef.	S.E.	Coef.	S.E.	Coef.	S.E.	Coef.	S.E.	Coef.	S.E.	Coef.	S.E.
Within individual effects														
Time	0.032**	0.012	0.028^**^	0.011	0.026^**^	0.011	0.026^**^	0.010	0.026^*^	0.010	0.027^**^	0.011	0.024^**^	0.010
Resistance friends			0.115^**^	0.020	0.117^**^	0.020	0.158^**^	0.022	0.079	0.054	0.116^**^	0.020	0.118^**^	0.020
Resistance non-friends (nf)					0.120^**^	0.052	0.166^**^	0.051	0.111^*^	0.050	0.303	0.229		
Resistance male nf													0.069^+^	0.040
Resistance female nf													0.119^**^	0.037
Status adolescent	−0.002	0.004	0.000	0.004	0.001	0.004	0.000	0.004	−0.000	0.004	0.001	0.004	0.001	0.004
Status friends	−0.012^**^	0.004	−0.024^**^	0.004	−0.024^**^	0.004	−0.024^**^	0.004	−0.019^**^	0.004	−0.024^**^	0.004	−0.024^**^	0.004
Status non-friends	−0.018	0.016	−0.022	0.014	−0.020	0.013	−0.020	0.013	−0.020	0.013	−0.020	0.013	−0.019	0.013
Proportion non-befriended boys	0.118	0.133	0.134	0.128	0.109	0.129	0.095	0.124	0.087	0.133	0.123	0.124	0.142	0.128
Between individual effects														
Boy	0.125^**^	0.022	0.106^**^	0.016	0.093^**^	0.025	0.093^**^	0.025	0.138 ^a **^	0.035	0.008^b^	0.065	0.121	0.026
Resistance friends			0.552^**^	0.024	−0.625^**^	0.053	−0.625^**^	0.053	−0.623^**^	0.053	−0.625^**^	0.053	0.044	0.041
Resistance non-friends (nf)					−5.725^**^	0.247	−5.725^**^	0.247	−5.715^**^	0.247	−5.732^**^	0.246		
Resistance male nf													−1.350^**^	0.066
Resistance female nf													−1.242^**^	0.080
Status adolescent	−0.013^*^	0.005	−0.003	0.005	−0.003	0.006	−0.003	0.006	−0.003	0.006	−0.003	0.006	−0.013	0.005
Status friends	−0.057^**^	0.007	−0.103^**^	0.007	0.064^**^	0.016	0.064^**^	0.016	0.070^**^	0.017	0.063^**^	0.016	−0.064^**^	0.011
Status non-friends	−0.022	0.017	−0.008	0.012	0.100	0.088	0.100	0.088	0.096	0.088	0.079	0.091	−0.164^*^	0.049
Proportion non-befriended boys	−0.117	0.086	0.001	0.052	0.853^**^	0.176	0.853^**^	0.176	0.793^**^	0.188	0.854^**^	0.176	0.273^+^	0.139
Parental education	−0.029^**^	0.006	−0.023^**^	0.005	−0.011^**^	0.004	−0.011^**^	0.004	−0.011^**^	0.004	−0.011^**^	0.004	−0.016^**^	0.005
Interactions														
Boy * time	0.029^+^	0.018	0.030^+^	0.017	0.030^+^	0.017	0.030^+^	0.017	0.023	0.016	0.028	0.017	0.032^*^	0.016
Boy * resistance friends (w.i.)							−0.082^*^	0.036	−0.225^**^	0.081				
Boy * resistance non-friends (w.i.)							−0.097	0.085			−0.411	0.284		
Boy * status friends (b.i.)									−0.014	0.009				
Boy * status non-friends (b.i.)											0.035	0.026		
Status friends (b.i.) * resistance friends (w.i.)									0.026^+^	0.016				
Boy * status friends (b.i.) * resistance friends (w.i.)									0.049^*^	0.024				
Boy * resistance male nf (w.i.)													−0.027	0.052
Boy * resistance female nf (w.i.)													−0.064	0.070
Status non-friends (b.i.) * resistance non-friends (w.i.)											−0.060	0.093		
Boy * status non-friends (b.i.) * resistance non-friends (w.i.)											0.145	0.125		
Variance components														
Class-level variance	0.018	0.003	0.000	0.000	1.878	0.313	1.878	0.313	1.868	0.312	1.879	0.314	0.388	0.054
Individual-level variance	0.238	0.009	0.218	0.086	0.095	0.006	0.095	0.006	0.095	0.006	0.095	0.006	0.154	0.007
Time variance	0.119	0.004	0.117	0.004	0.117	0.004	0.117	0.004	0.116	0.004	0.117	0.004	0.117	0.004

^a^ The main effect of ‘boy’ in this models is not comparable to the ‘boy’ effect in the other models. The ‘boy’ effect in this model refers to the effect of being a boy when ‘status friends’ at the between-individual level is 0. In models 1–4 the ‘boy’ effect refers to the effect of being a boy when ‘status friends’ at the between-individual level is at its mean level. This mean level is not 0. In the current model, the ‘boy’ effect is 0.089 when the ‘status friends’ at the between-individual level is at its mean level

^b^ The main effect of ‘boy’ in this models is not comparable to the ‘boy’ effect in the other models. The ‘boy’ effect in this model refers to the effect of being a boy when ‘status non-friends’ at the between-individual level is 0. In models 1–4 the ‘boy’ effect refers to the effect of being a boy when ‘status non-friends’ at the between-individual level is at its mean level. This mean level is not 0. In the current model, the ‘boy’ effect is 0.093 when the ‘status non-friends’ at the between-individual level is at its mean level

^+^<0.10, *<0.05, **<0.01 (two-sided tests)

In model 2, we add the resistant behavior of friends. We find that a one-unit increase in the resistance of friends is related to a 0.115 increase in adolescents’ resistance to schooling (supporting hypothesis 1b). Compared to model 1, the time-stable gender difference in resistance to schooling is reduced by 15% (i.e., (1-(0.106/0.125)*100).^7^ However, the increase in the gender gap over time is not reduced. Hence, we find limited support for hypothesis 1.

Model 3 adds the behavior of non-friends in class. A one-unit increase in the resistant behavior of non-befriended classmates is related to a 0.120 increase in the resistant behavior of the respondent,^8^ very similar to the effect of friends.

In model 4, we test whether the resistant behavior of friends and non-friends, respectively, is more positively related to the resistant behavior of boys than that of girls (hypothesis 2a and 2b).^9^ Our results are in fact contrary to these assumptions. For girls, a one-unit increase in the resistant behavior of friends is related to a 0.158 increase in their resistance to schooling (*p* < 0.001), while the corresponding figure for boys is 0.077 (*p* = 0.012). The interaction between gender and the resistant behavior of non-friends is also negative, and the point estimate of similar size, but not statistically significant. Compared to the previous model without the interaction effects, the (increase in the) gender gap is not altered.

Next, we examine whether boys are more influenced than girls by the resistant behavior of high-status friends (model 5) and non-friends (model 6). In model 5, the interaction between the between-individual effect of the social status of friends and the within-individual effect of the resistant behavior of friends is positive and borderline significant. This indicates that an increase in the resistant behavior of friends is more positively related to an increase in the adolescent’s resistant behavior when the average social status of friends is higher. In line with hypothesis 3a, this is more so for boys than for girls (i.e., the three-way interaction between gender, the resistant behavior of friends, and the social status of friends is positive and significant).

**Fig. 2:**
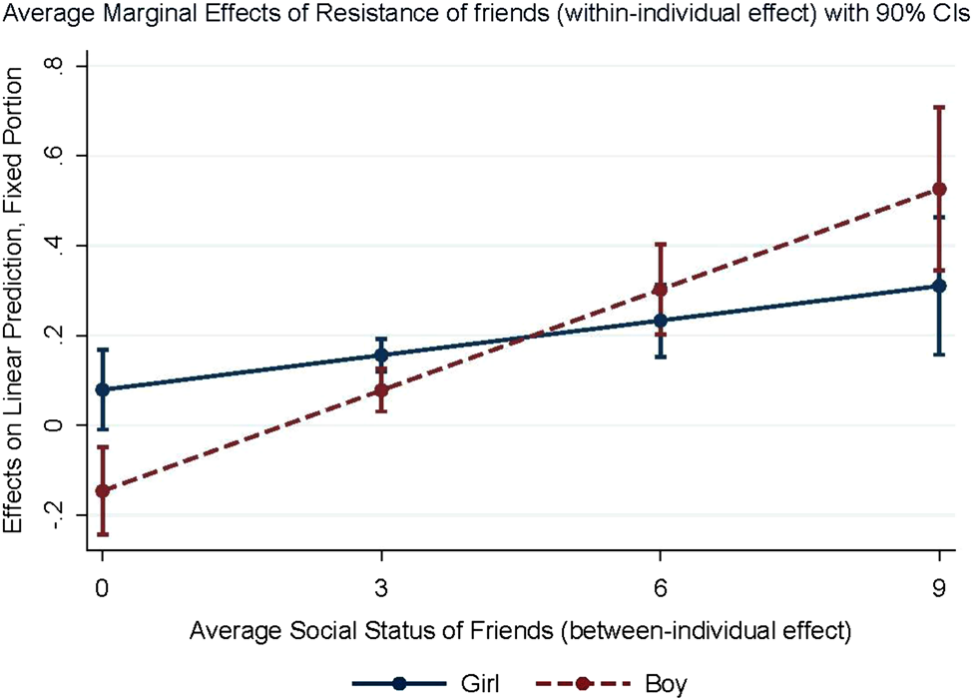
Average marginal effect of resistance of friends by the social status of friends and gender

To shed more light on this three-way interaction effect, we plot the average marginal effects of the resistant behavior of friends for different values of friends’ social status for boys and girls. Figure 2 indicates that when the average social status of friends is low (about two incoming friendship ties or less), an increase in their resistant behavior is more positively related to an increase in the resistant behavior of girls than that of boys. The corresponding gender difference in the influence of friends with a higher social status is well covered by the confidence intervals.

The average social status of friends is positively correlated with their average resistant behavior (0.2 in wave 1 and 0.4 in wave 2). The significant three-way interaction thus implies that, compared to girls, boys are less influenced by friends who tend to exhibit low levels of resistance to schooling. This accounts for some of the increase in the gender gap over time. Compared to the previous model, the increase in the gender gap (i.e. the boy*time interaction) is reduced by 23% (from 0.030 to 0.023).^10^ We do not find statistically significant interactions between the social status of non-friends, the resistant behavior of non-friends and gender (hypothesis 3b; model 6).

Finally, we test whether the resistant behavior of same-sex non-friends is more positively related to adolescents’ school resistance than the resistant behavior of non-friends’ opposite-sex classmates (model 7, hypothesis 4). For girls the results are in line with the hypothesis. A one-unit increase in resistant behavior of male non-friends is related to a 0.068 increase in the resistant behavior of girls, while the corresponding effect of female non-friends is 0.119. However, the difference in the sizes of these two relationships is statistically insignificant (F-test (1, 0) = 0.77, *p* = 0.379). For boys, neither the same-sex effect of 0.042 (*p* = 0.207) nor the opposite-sex effect of 0.055 (*p* = 0.334) is statistically significant, which also is true of the difference between these estimates (F-test (1, 116.9) = 0.03, *p* = 0.856).

### SIENA Models

Table 4 shows the results of the SIENA analyses on students’ resistance to schooling and their friendships in class. The estimates are presented in log-odds-ratio’s (Ripley et al. 2017). Model 1 indicates that the odds for boys to increase their resistance to schooling (rather than not) is 1.267 (OR = *e* ^0.237^ = 1.267) greater than the corresponding odds for girls.

**Table 4:** SIENA meta-analyses on 18 multiple group analyses on adolescents’ friendships networks and their resistance to schooling in Sweden. *N* individuals = 2480; *N* school classes = 98

	Model 1			Model 2			Model 3			Model 4		
	Coef.	S.E.	Fisher test	Coef.	S.E.	Fisher test	Coef.	S.E.	Fisher test	Coef.	S.E.	Fisher test
Network formation												
Outdegree	**−4.545** **	**0.633**	**FL**	**−4.447** **	**0.616**	**FL**	**−**4.264**	0.063	FL	**−**4.289^**^	0.651	FL
Reciprocity	1.572**	**0.067**	**FR**	1.562**	**0.065**	**FR**	1.566**	**0.065**	**FR**	**1.568** ^**^	**0.065**	**FR**
Transitive triplets	**0.556** **	**0.025**	**FR**	**0.558** **	**0.024**	**FR**	**0.556** **	**0.024**	**FR**	0.558^**^	0.024	FR
3-cycle	**−**0.463^**^	0.053	FL	**−0.461** **	**0.053**	**FL**	**−**0.463**	0.052	FL	**−**0.466^**^	0.054	FL
Outdegree activity	**−0.072**	**0.169**		**−0.076**	**0.164**		**−0.036**	**0.167**		**−**0.018	0.172	
Outdegree activity sqrt	**1.173** ^**+**^	**0.640**		**1.151** ^**+**^	**0.621**		**0.972**	**0.633**		0.935	0.654	
Resistance alter	0.000	0.019		0.000	0.018		0.002	0.018		0.001	0.002	
Resistance ego	**0.017**	**0.027**		0.026	0.028		**0.023**	**0.029**		0.019	0.026	
Resistance homophily	0.383^*^	0.137	FR	0.365^*^	0.138	FR	0.366*	0.136	FR	0.387^*^	0.140	FR
National-Origin homophily	0.178^+a^	0.089	FR	0.189^*a^	0.088	FR	0.182^*+* a^	0.089	FR	0.184^*+* a^	0.088	FR
Native alter	**−**0.136^+a^	0.071	FL	**−**0.136^+a^	0.071	FL	**−**0.136^*+* a^	0.071	FL	**−**0.129^*+* a^	0.071	FL
Native ego	0.139^a^	0.094	FR	0.133^a^	0.093	FR	0.137^a^	0.095	FR	0.134^a^	0.094	FR
Boy alter	**−**0.009	0.043		**−**0.011	0.043		**−**0.012	0.043		**−**0.011	0.042	
Boy ego	0.057^a^	0.090	FR	0.055^a^	0.089	FR	0.061^a^	0.090	FR	0.049^a^	0.091	FR
Sex homophily	0.430^**^	0.047	FR	0.430^**a^	0.047	FR	0.429**	0.047	FR	0.428**	0.047	FR
Behavior												
Linear shape	**−0.004**	**0.128**		**−0.022**	**0.133**		**−**0.022	0.130		0.003	0.135	
Quadratic shape	**−0.050** ^*^	**0.024**	**FL**	−0.074*	**0.026**	**FL**	**−**0.067*	0.026	FL	**−0.066** *	**0.026**	**FL**
Resistance friends				**0.232** **	**0.066**	**FR**	**0.234** **	**0.064**	**FR**	0.221**	**0.065**	**FR**
Boy	0.237^**^	0.065	FR	**0.217** **	**0.071**	**FR**	**0.218** **	**0.071**	**FR**	**0.217** **	**0.071**	**FR**
Parental education	**−0.036**	**0.025**	**FL**	**−0.030**	**0.025**		**−0.027**	**0.024**		**−0.025**	**0.025**	
Status friends	**−**0.008	0.029		**−**0.008	0.030		**−**0.008	0.029		**−**0.013	0.030	
Boy * resistance friends^b^							N.A.	N.A.		N.A.	N.A.	
Boy * status friends^b^										N.A.	N.A.	
Status friends * resistance friends^b^										N.A.	N.A.	
Boy* status friends * resistance friends^b^										N.A.	N.A.	

*Note*: Estimates and s.e.’s are obtained according to the Snijders and Baerveldt (2003) method;When the estimates and s.e.’s are printed bold, the correlation between the estimate and the s.e. is significant, and we rely on Fisher type tests. FL= P-value of Fisher’s left sided test < 0.025; FR = P-value of Fisher’s right sided test < 0.025

^a^ The estimate significantly varies across the 18 groups

^b^ Interaction terms are score tested. The Fisher-test column indicates whether either Fisher’s right-sided (FR) or Fisher’s left sided test (FL) are <0.05

^+^<0.10, *<0.05, **<0.01 (two^-^sided tests)

We hypothesized that boys are more exposed to resistant behavior in their friendship group (hypothesis 1a, which was supported by t-tests), because adolescents tend to befriend same-sex peers and peers who exhibit similar levels of resistant behavior. We do find positive and significant gender homophily effects and resistance homophily effects in all the SIENA models, supporting the idea that adolescents tend to befriend classmates of the same sex and who engage in similar levels of resistance to schooling.

In model 2 we find that the resistant behavior of friends positively and significantly influences the resistant behavior of adolescents, supporting hypothesis 1b. When friends score one point higher on student resistance, adolescents’ odds of increasing their resistant behavior are 1.261 higher than their odds of maintaining their initial level (*e* ^0.232^). When accounting for the effect of friends’ resistant behavior, the boy effect reduces slightly, and remains statistically significant. While we cannot conclude that there is no mediation (see page 18; and Mood 2010), the results suggest that the resistant behavior of friends does not fully account for the gender gap in the increase in resistance to schooling.

Is there a gender difference in the responsiveness to the resistant behavior of friends (hypothesis 2a)? Score type tests show that the interaction between gender and the resistant behavior of friends in model 3 is not statistically significant (χ^2^(36) = 30.705, *p* = 0.718; left-sided score-type test: χ^2^(36) = 46.011, *p* = 0.123).^11^ This finding differs from the findings of the hybrid model (Table 3), which is probably because we use a restricted sample for the SIENA analyses (the interaction between gender and the resistant behavior of friends is not statistically significant in a hybrid model on the SIENA sample (see Appendix A4)).

Finally, we test whether the influence of the resistant behavior of friends with a higher social status is stronger for boys than for girls (hypothesis 3a). It appears not to be, as the interaction between the social status and resistant behavior of friends is not statistically significant (right-sided score-type test χ^2^(32) = 31.128, *p* = .510; left-sided score-type test: χ^2^(36) = 33.235, *p* = .407). Moreover, we find no statistically significant three-way interaction between gender, the social status of friends, and the resistant behavior of friends (right-sided score-type test χ^2^(32) = 37.002, *p* = .249; left-sided score-type test: χ^2^(32) = 36.280, *p* = .276). Again, hybrid models on the SIENA sample indicate that this may be due to our restricted sample, as we find no support for these interactions in the hybrid models on the SIENA sample (see Appendix A4).

#### Goodness of fit tests and robustness checks of the SIENA model

We assess the Goodness of fit (GoF) of the SIENA models with a method that uses auxiliary statistics (see Appendix A5). In 92 classes or more, the model fit was adequate for the indegree, the geodesic distance, and resistance to schooling. Outdegree and transitive ties seemed to be modelled inadequately in more classes (see Appendix A5). We estimate several additional models to improve the model fit for these statistics (see Appendix A5 and A6). Some of these model modifications improve the model fit for some classes, yet they worsen the fit for others. Reassuringly, however, modifications to the specifications of the SIENA model do not alter our main findings (see Appendix A6).

## Discussion

Previous research has indicated that boys exhibit more resistance to schooling than girls (for reviews on the gender gap in school outcomes, see: Buchmann et al. 2008; Driessen and van Langen 2013). While scholars have highlighted the role of peer processes in this gender gap, there are relatively few quantitative studies that actually test the role of peers, and existing quantitative research has been limited to cross-sectional data. This study contributed to past research by explicitly and longitudinally studying the role of peer processes in gender differences in student resistance to schooling. We hypothesized that gender differences in both the exposure and the response to resistant peers may lead to time-stable or increasing gender differences in resistant behavior. We estimated hybrid models on panel data on more than 5000 adolescents (age 14–15 in wave 1) in over 200 school classes in Sweden. On a subsample of the data, we employed novel statistical social network techniques. We found that, overall, boys show more resistance to schooling than girls, and that the gender gap slightly widened across a one-year-period.

The findings suggested that, compared to girls, boys are more exposed to friends who exhibit resistance to schooling. Moreover, and importantly, adolescents seemed to be positively influenced by the resistant behavior of their friends. These peer selection and peer influence processes did not account for the widening gender gap in resistance to schooling over time. However, our results indicated that they contributed somewhat to the persistence of the initial gender gap. It could be that boys are influenced to behave similarly to the average behavior of their male friends, and girls are influenced to behave similarly to the average behavior of their female friends, which would lead to time-constant gender differences in resistant behavior. Gender differences in resistance to schooling may possibly be less persistent over time if boys and girls would befriend different (e.g., opposite-sex) peers.

We did not find that boys responded more to their friends’ resistant behavior than girls. Instead, our results suggested that girls were more influenced by the average behavior of their friends than boys, seemingly due to the fact that girls were more positively influenced by the resistant behavior of low-status friends. Boys’ emulation of the resistant behavior of friends may be motivated by their desire to gain status, and therefore they tend not to be influenced by low status friends. Girls may be influenced by the resistant behavior of friends for other reasons, such as the maintenance of friendship ties. The fact that, compared to girls, boys were less influenced by the resistant behavior of low status friends somewhat explained why boys increased their resistant behavior more than girls.

We did not find that girls and boys differed in their responsiveness to the behavior of non-befriended classmates. Relatedly, we did not find that students’ school resistance was influenced more by the resistant behavior of same-sex non-friends than that of opposite-sex non-friends. In general, peer processes did not seem to account for much of either the time-stable or the increasing gender gap in resistance to schooling over time. Although our findings should be repeated on other data in different countries, they are potentially of great theoretical importance. Policies that aim to tackle gender differences in educational outcomes by focusing on gendered peer processes related to school resistance may, according to our results, only have limited effects.

This study also knows some limitations. First, the evolution of resistant behavior and the formation of friendships are interdependent processes. Although our SIENA models handled the feedback processes between friendship selection and the influence of friends, we could not fit a corresponding model for the feedback processes between friendship selection and the influence of non-befriended classmates. In the hybrid models, we were unable to rule out that a change in student resistance was (also) related to a change in the resistance of (non-)befriended classmates, rather than the other way around. This is related to the fact that we only had two waves of data. With more observation points, we could have shed more light on the temporal ordering of the relationships in the hybrid models. This would have also allowed us to examine whether boys consistently increase their resistance to schooling more than girls.

Second, and relatedly, our data pertain to a particular age (mainly 14–15), and a particular observation window (1 year), and we must be careful to generalize over and above those. Longitudinal school (network) data over longer periods of time are however scarce, one reason being that school classes often change their composition, and previous studies have also encountered this problem. In fact, although our data had shortcomings, they were still unique. Network studies are often based on case studies (e.g., in one school). This study included schools representing the whole of Sweden, making it much more possible to draw inferences to the population of adolescents. Moreover, we studied adolescents at a crucial period in their development in which deviant behavior is around its peak (Moffitt 1993), and during which peers act as central socializing agents (Veenstra et al. 2013).

## Conclusion

Scholars have frequently argued that peers play a pivotal role in the gender gap in resistance to schooling (Driessen and Van Langen 2013), and the subsequent under-achievement and attainment of boys in school (Hadjar and Buchmann 2016). Boys would be pressured in their peer groups to be defiant in class, as this would lead to higher social status and prevent them from being ostracized. The idea that peers play a significant part in boys’ resistance to schooling is mainly based on small-scale qualitative studies, or cross-sectional quantitative studies. However, peer processes are dynamic, and can only be studied adequately with longitudinal data. In this research we were able to overcome this lacunae by explicitly studying the role of peers for gender differences in student resistance, using large-scale panel data for Sweden. Our analyses supported the hypothesis of peer influences on school resistance. At the same time, our study somewhat nuanced the role of peers for boys’ greater resistance to schooling, as we found that peer processes contributed only slightly to this gender gap and increases herein.

## Appendix

**A1 Information on the English, German, and Dutch data: reasons for exclusion and the gender gap**

In the Netherlands, student mobility across school classes is very high, especially for students in the higher tracks. On average 52% of a Dutch respondent’s classmates in the first wave are not part of his/her class in the second wave. The opportunity structure for friendships in class thus changes over time, and changes in the behavior of friends and/or non-friends may purely stem from changes in the class composition. All in all, none of the Dutch class networks are suitable for longitudinal social network analyses (Ripley et al. 2017; CILS4EU 2016).

In England, the school class is not the prime context for educational activities and friendship formation in school. Students tend to move to different classes for different subjects, and only 41% of all friendships in school are friendships to classmates in the first wave of the data collection.^12^ Hence, peer processes outside of class are likely to play a more prominent role in the gender gap in resistance to schooling in England, and a study on peer processes in class would therefore be misleading. Moreover, about 80% of the English class networks are unsuitable for longitudinal social network analyses (see CILS4EU 2016).^13^

In 18% of the German schools, students left their school after the first wave, as they finished their education. This implies that we only have longitudinal information on resistance to schooling and friendship networks in class for a selective German sample, as students who finished school after the first wave are students from low ability tracks. Students from lower ability tracks generally exhibit higher levels of resistance to schooling (Dumont et al 2017). Besides this issue, about 75% of the German classes are unsuitable for longitudinal social network analyses (CILS4EU 2016).

While gender differences in resistance to schooling are apparent in Sweden, they tend to be larger in the other countries in the CILS4EU data. Compared to Sweden, the gender gap in resistance to schooling is significantly larger in Germany in the first wave (1.9 times larger), and larger in all the other three countries in the second wave (1.6 times larger in Germany; 1.9 times larger in England; 1.5 times larger in the Netherlands).^14^ Nevertheless, there are no significant country differences in the gender gap in the increase in resistance to schooling over time.

**A2 Representativeness of the analytical sample**

We use two sample t-tests to examine whether students that were included in the SIENA analyses significantly differ from students that were excluded with respect to resistance to schooling. For these test we use the average score on the resistance to schooling items, and not the ordinal variable that is used in the SIENA analyses. Compared to students that are excluded from the SIENA sample, students that are included in the sample score 0.074 points lower on resistance to schooling in the first wave, and 0.039 points lower in the second wave. These differences are statistically significant (wave 1:t(5003) = 4.311, *p* *<* 0.001; wave 2:t(4786) = 2.245, *p* = 0.025). Little’s MCAR tests show that students resistance to schooling is not missing completely at random in the sample we use for the SIENA analyses (Little χ^2^(2) = 62.344, *p* *<* 0.001). Moreover, students are also not missing completely at random in the full sample (Little χ^2^(2) = 160.166, *p* *<* 0.001). We have to be aware of this when drawing conclusions.

**A3 Test of the ‘equality of parameters’ assumption of the SIENA multi-group models**

We combine several class networks in a multi-group analysis in SIENA (i.e., with the sienaGroupCreate function). The analyses take into account that adolescents can only befriend students who attend their own class

By combining multiple classes in one big network the power and convergence of the models is improved. However, multi-group models assume that parameters^15^ are the same in the different classes that we combine in one analysis. We test this assumption for the hypothesized effects with the sienaTimeTest function for model 2–4 in Table 4. For the behavioral part of the analyses these effects are: the effect of the resistant behavior of friends, the boy effect, the interaction between boy and the resistant behavior of friends, and the interaction between the boy, social status of friends, and the resistant behavior of friends. For the friendship formation part of the model, we test this assumption for the gender homophily and resistant behavior homophily effect.

The ‘equality of parameter’ assumption is met for the hypothesized effects in 10 of the 18 groups. In the groups for which the assumption is not met, we delete classes that violate the assumption and rerun the multiple group models and the meta-analyses until the assumption is met for all hypothesized effects.

The results (full tables available upon request) are highly similar to the results reported in Table 4, and in line with our main conclusions. In line with the results reported in the main text, we still find support for gender homophily and resistant behavior homophily effect in models 2–4. Moreover, we still find support for a positive effect of the resistant behavior of friends and a positive effect of being a boy on the evolution of resistant behavior in models 2–4. In model 3, the left-sided score type test for the interaction between gender and the resistant behavior of boys is borderline insignificant^16^ (Left one-sided score type test: χ^2^ = 45.505, *p* = 0.090; Right-one-sided test Fisher type tests χ^2^ = 31.695, *p* = 0.583). A significant left-one sided test would indicate that, compared to girls, boys are less likely to increase their resistance to schooling when their friends exhibit higher levels of resistance to schooling. The three-way interaction that is tested in model 4 is not significant (Left one-sided score type test: χ^2^ = 36.280, *p* = 0.276; Right-one-sided test Fisher type tests χ^2^ = 37.002, *p* = 0.249). The left-sided score type tests for the interaction between the resistant behavior of friends and gender is again borderline insignificant (Left one-sided score type test: χ^2^ = 42.823, *p* = 0.096; Right-one-sided test Fisher type tests χ^2^ = 33.337, *p* = 0.402)

**A4 Hybrid models on the SIENA sample**

See Table 5.

**Table 5:** Hybrid models on resistance to schooling (SIENA sample). *N* classes = 98; *N* students = 2273*; N* obs. = 4536

	Model 1		Model 2		Model 3		Model 4		Model 5		Model 6		Model 7	
	Coef.	S.E.	Coef.	S.E.	Coef.	S.E.	Coef.	S.E.	Coef.	S.E.	Coef.	S.E.	Coef.	S.E.
Within individual effects														
Time	0.028	0.018	0.025	0.016	0.017	0.016	0.018	0.016	0.017	0.016	0.019	0.017	0.017	0.016
Resistance friends			0.111^**^	0.031	0.117^**^	0.030	0.136^**^	0.037	0.062	0.094	0.118^**^	0.029	0.119^**^	0.030
Resistance non-friends (nf)					0.175^*^	0.077	0.138^+^	0.081	0.161^*^	0.075	0.266	0.534		
Resistance male nf													0.097	0.068
Resistance female nf													0.081	0.062
Status adolescent	−0.004	0.006	−0.002	0.006	−0.002	0.006	−0.002	0.006	−0.001	0.006	−0.002	0.006	−0.002	0.006
Status friends	−0.005	0.008	−0.015^*^	0.007	−0.017^*^	0.007	−0.017^*^	0.007	−0.012^+^	0.007	−0.017^*^	0.007	−0.017^*^	0.007
Status non-friends	−0.004	0.023	−0.012	0.022	−0.010	0.019	−0.010	0.019	−0.007	0.019	−0.010	0.019	−0.009	0.020
Proportion non-befriended boys	0.243	0.181	0.275	0.180	0.267	0.178	0.250	0.178	0.250	0.178	0.267	0.178	0.267	0.177
Between individual effects														
Boy	0.088^*^	0.034	0.070^**^	0.024	0.040	0.037	0.041	0.037	0.110^+a^	0.064	−0.027^b^	0.121	0.052^*^	0.024
Resistance friends			0.605^**^	0.036	−0.641^**^	0.097	−0.641^**^	0.097	−0.639^**^	0.097	−0.641^**^	0.097	0.137^**^	0.052
Resistance non-friends (nf)					−6.303	0.450	−6.303	0.450	−6.297	0.449	−6.308	0.451		
Resistance male nf													−1.259	0.111
Resistance female nf													−1.140	0.119
Status adolescent	−0.016^*^	0.007	−0.008	0.007	0.000	0.008	0.000	0.008	−0.001	0.008	0.000	0.008	−0.016^*^	0.007
Status friends	−0.057^**^	0.012	−0.094^**^	0.011	0.048^*^	0.020	0.048^*^	0.020	0.023	0.027	0.048^*^	0.020	0.077^**^	0.015
Status non-friends	−0.078^*^	0.037	−0.047^*^	0.023	0.048	0.094	0.048	0.094	0.040	0.050	0.034	0.096	−0.233^**^	0.077
Proportion non-befriended boys	−0.175	0.117	−0.027	0.079	0.595	0.257	0.595^*^	0.258	0.509^+^	0.280	0.588^*^	0.255	0.207	0.206
Parental education	−0.030^**^	0.008	−0.016^*^	0.007	−0.012^+^	0.006	−0.012^+^	0.006	−0.011^+^	0.006	−0.012^+^	0.006	−0.015^*^	0.007
Interactions														
Boy * time	0.059^*^	0.025	0.057^*^	0.024	0.057^*^	0.024	0.055^*^	0.025	0.048^*^	0.023	0.054^*^	0.026	0.052^*^	0.024
Boy * resistance friends (w.i.)							−0.035	0.056	−0.206	0.168				
Boy * resistance non-friends (w.i.)							0.069	0.143			−0.057	0.763		
Boy * status friends (b.i.)									−0.020	0.017				
Boy * status non-friends (b.i.)											0.025	0.043		
Status friends (b.i.) * resistance friends (w.i.)									0.023	0.027				
Boy * status friends (b.i.) *resistance friends (w.i.)									0.052	0.048				
Boy * resistance male nf (w.i.)													0.028	0.089
Boy * resistance female nf (w.i.)													−0.046	0.109
Status non-friends (b.i.) * resistance non-friends (w.i.)											−0.047	0.198		
Boy * status non-friends (b.i.) * resistance non-friends (w.i.)											0.048	0.298		
Variance components														
Class-level variance	0.015	0.003	0.000	0.000	1.745	0.303	1.745	0.303	1.740	0.303	1.748	0.304	0.298	0.066
Individual-level variance	0.214	0.012	0.190	0.167	0.078	0.008	0.079	0.008	0.079	0.008	0.079	0.008	0.141	0.009
Time variance	0.107	0.006	0.107	0.109	0.106	0.006	0.106	0.006	0.105	0.006	0.106	0.006	0.106	0.006

The sample size is smaller than the sample size in the SIENA models, as students who did not participate in both waves are excluded here. In the SIENA models these students are part of the network, as they could be nominated by others

^a^ The main effect of ‘boy’ in this models is not comparable to the ‘boy’ effect in the other models. The ‘boy’ effect in this model refers to the effect of being a boy when ‘status friends’ at the between-individual level is 0. In models 1–4 the ‘boy’ effect refers to the effect of being a boy when ‘status friends’ at the between-individual level is at its mean level. This mean level is not 0. In the current model, the ‘boy’ effect is 0.033 when the ‘status friends’ at the between-individual level is at its mean level

^b^ The main effect of ‘boy’ in this models is not comparable to the ‘boy’ effect in the other models. The ‘boy’ effect in this model refers to the effect of being a boy when ‘status non-friends’ at the between-individual level is 0. In models 1–4 the ‘boy’ effect refers to the effect of being a boy when ‘status non-friends’ at the between-individual level is at its mean level. This mean level is not 0. In the current model, the ‘boy’ effect is 0. 0.041when the ‘status non-friends’ at the between-individual level is at its mean level

^+^<0.10, *<0.05, **<0.01 (two-sided tests)

**A5 Goodness of fit tests of the SIENA model**

We assess the Goodness of fit (GoF) of the SIENA model with a method that uses auxiliary statistics. We use model 2 to assess the GoF. Networks are simulated on the basis of the parameters in this SIENA model. The simulated networks are compared to the observed data with respect to several auxiliary statistics. More specifically, we compare the simulated network data with respect to four auxiliary network statistics—outdegree distribution, indegree distribution, geodesic distance, and triad census—and one auxiliary behavior statistic—the behavior distribution of resistance to schooling. A significant statistic indicates that the effects in the SIENA model do not adequately represent friendship or behavioral patterns in the observed data. This may indicate that additional effects should be included in the model. Statistics are calculated for each of the 98 classes (see Table 6). As we test the same hypothesis multiple times, we use the Bonferroni correction for multiple testing (also see Block 2015). More specifically, we take a significance level of α/n. α is 0.05 and n is the number of classes in the multiple group model. When the p-value *<* α/n, the multiple group model fit is inadequate, and the p-value is printed bold in Table 6.

**Table 6:** P-values of the goodness of fit statistics for the SIENA model

Group	Class	Resistance to schooling	Indegree	Outdegree	Geodesic distance	Triad census
**1**	1	**<0.001**	0.169	0.176	0.483	0.342
	2	0.428	0.134	0.711	0.143	0.074
	3	0.741	0.995	**0.004**	0.075	0.213
**2**	4	0.101	0.807	0.799	0.011	0.201
	5	0.143	0.754	0.011	0.124	0.540
	6	0.048	0.800	0.145	0.967	**0.008**
	7	0.116	0.372	0.301	0.403	0.108
	8	0.396	0.213	0.025	0.304	0.546
	9	0.535	0.475	0.010	0.227	0.464
**3**	10	**0.001**	0.071	0.049	0.231	0.799
	11	0.205	0.658	0.895	0.949	1.000
	12	0.118	0.511	0.051	0.430	0.165
	13	0.834	0.328	0.031	0.428	0.033
**4**	14	**<0.001**	0.038	0.027	0.393	0.080
	15	0.754	0.472	0.632	0.333	0.410
	16	0.176	0.881	0.864	0.292	0.878
	17	0.239	0.750	0.591	**0.003**	0.381
	18	0.628	0.809	0.134	0.032	0.597
	19	0.556	0.463	**<0.001**	0.489	0.103
**5**	20	0.732	0.872	0.016	0.506	0.196
	21	0.094	0.271	**0.001**	0.051	0.097
	22	0.652	0.309	0.018	0.209	0.027
	23	0.139	0.099	0.070	**0.002**	**<0.001**
	24	0.777	0.451	0.482	0.844	0.239
**6**	25	0.516	0.545	0.056	0.948	0.145
	26	0.770	0.428	0.461	0.760	0.047
	27	0.063	0.413	0.023	0.043	0.017
	28	0.600	0.800	0.189	0.351	0.103
	29	**0.183**	0.772	0.261	0.556	0.293
**7**	30	**0.387**	0.752	0.036	0.032	0.086
	31	0.268	0.842	**0.002**	0.059	0.468
	32	0.086	0.552	**0.003**	0.573	0.956
	33	0.025	0.250	0.517	0.029	0.173
	34	0.342	0.910	0.869	0.042	0.338
	35	0.202	0.014	0.100	0.469	0.101
8	36	**0.004**	0.594	0.100	0.039	**0.009**
	37	0.694	**0.004**	0.025	0.055	0.011
	38	0.497	0.056	0.071	0.840	0.189
	39	0.101	0.787	**0.008**	0.146	0.484
	40	0.704	0.435	0.065	0.333	0.383
9	41	0.308	0.363	**<0.001**	0.531	0.232
	42	0.178	0.139	0.056	0.475	0.017
	43	0.096	0.435	0.220	0.224	0.628
	44	0.397	0.687	0.554	0.043	0.071
	45	0.767	0.088	0.676	0.256	0.416
	46	0.594	0.238	0.316	0.120	0.089
10	47	0.442	0.563	0.135	0.108	0.011
	48	0.282	0.301	0.673	0.489	0.850
	49	0.499	0.371	0.616	0.160	**0.001**
	50	0.030	0.479	0.027	0.841	0.279
	51	0.781	0.095	0.319	0.824	0.367
	52	0.399	0.233	0.012	0.843	0.321
11	53	0.751	0.767	0.018	0.849	0.401
	54	0.040	0.096	**0.006**	**0.005**	0.029
	55	0.530	0.222	0.009	0.735	0.037
	56	0.056	0.439	0.661	0.625	0.054
	57	0.535	0.215	0.028	0.498	0.914
	58	0.678	0.237	0.212	**0.004**	**0.005**
12	59	0.025	0.548	**0.006**	0.333	0.014
	60	0.272	0.800	0.410	0.097	**0.001**
	61	0.471	0.294	0.043	**0.007**	0.226
	62	0.756	0.070	0.111	0.269	0.041
	63	0.024	0.367	**0.007**	**0.007**	0.019
		0.410	0.571	0.031	0.302	0.198
13	65	0.063	0.412	0.196	0.048	0.541
	66	0.690	0.589	0.010	0.554	0.681
	67	0.983	0.164	**0.001**	0.011	0.210
	68	0.851	0.786	0.171	0.271	0.115
	69	0.162	0.060	**0.001**	0.072	**0.001**
	70	0.189	0.309	**0.001**	0.616	**<0.001**
14	71	0.075	0.963	0.060	0.969	0.359
	72	0.243	0.528	0.049	0.276	0.299
	73	**0.005**	0.329	0.591	0.980	0.737
	74	0.154	0.818	0.581	0.019	0.726
	75	0.773	0.157	0.695	0.263	0.019
	76	0.187	0.465	**0.002**	0.193	0.228
15	77	0.578	0.605	0.181	0.989	0.277
	78	0.383	0.558	**0.004**	0.032	0.521
	79	0.073	0.104	0.804	0.649	0.666
	80	0.889	0.561	0.024	0.552	0.318
	81	0.512	0.016	**0.003**	0.044	0.013
16	82	0.217	0.075	0.142	0.009	0.130
	83	0.338	0.274	0.042	0.492	0.833
	84	0.745	0.757	0.366	0.039	0.197
	85	0.129	0.236	0.245	0.037	0.111
	86	**<0.001**	0.319	**0.004**	0.084	0.020
	87	0.352	0.660	0.053	0.645	0.586
17	88	0.127	0.918	0.198	0.760	0.119
	89	0.793	0.714	0.284	0.205	0.689
	90	0.247	0.365	0.300	0.446	0.879
	91	0.329	0.386	0.172	0.175	0.197
	92	0.093	0.442	0.053	0.260	0.048
	93	0.402	0.844	0.038	0.717	**<0.001**
18	94	0.510	0.660	0.171	0.381	0.063
	95	0.469	0.113	**<0.001**	0.221	0.023
	96	0.422	0.520	**0.008**	0.025	0.065
	97	0.700	0.041	0.343	0.643	0.416
	98	1.000	0.354	**0.008**	0.371	0.084

Model is rejected if a P-value of one of the classes in a group *<* (0.05 / number of classes in a group). P-value is printed bold if this is the case

For resistance to schooling, indegree, and geodesic distance, the model fit seems adequate in most classes. Resistance to schooling has an inadequate fit for 6 classes in 6 groups. The findings of the behavioral part of the model are not altered when these groups are excluded from the meta-analysis. However, homophily with respect to resistance to schooling turns to insignificance in the friendship part of the model (0.242 (s.e. 0.143), *p* = 0.118). The indegree effect has an inadequate fit in one class in group 8. Excluding this group from the meta-analysis does not alter the conclusions. For the geodesic distance, the fit is inadequate for 6 classes in 4 groups. Again, conclusion remain unaltered when these groups are excluded from the meta-analyses.

The outdegree effect seems to be modeled inadequately for 20 classes in 13 of the 18 multi-group models. Plots indicate that in most classes this was due to an underestimation of students with a low outdegree in the model. Initially, the fit for outdegree was even worse, and therefore we included the outdegree activity and the outdegree activity sqrt effects. The conclusions of the model remained the same. Moreover, we tried several other model specifications (see Appendix A5). More specifically, we estimated a model with a truncated outdegree effect instead of the outdegree activity sqrt effect. The truncated outdegree effect models people’s tendency to have no outgoing ties. Adding this effect improves the fit for several classes, but worsens it for others. Moreover, conclusions are not altered by including this effect.

The triad census specifies whether possible relationships among three actors (i.e., triads) are well represented by the model. The triad census is modeled inadequately for 9 of the 98 classes in 8 of the 18 groups. We inspect the plots of the GOF of the triad census to examine which triadic relationships are misrepresented by our model. In several classes, the inadequate fit for the triad census statistic is due to a misrepresentation of closed triads with one reciprocated tie. In some classes, triads with one-directional ties between the actors were misrepresented. Finally, triads with no or only one tie were sometimes misrepresented. Hence, the inadequate modeling of the transitive census may be related to an inadequate modeling of outdegree. To improve the fit of the model, we first estimated a model with the truncated outdegree effect instead of the outdegree activity sqrt effect. Second, we estimated a model with the transitive ties effect, instead of the transitive triplets effect. Finally, we estimated models with an interaction between the transitive triplets effect and the reciprocity effect (i.e., the transRecTrip effect) (see Appendix A6). Some of these modifications—especially the last one—improve the model fit for the triad census statistic for several classes. However, it worsens the model fit for other classes. Moreover, the conclusions are not altered by these model modifications.

**A6 Alternative model specifications of the SIENA model**

We check whether the SIENA results are robust to several alternative model specifications. We apply several modification to model 2 in Table 4. Based on the Goodness of Fit results, we estimate models in which we use different effects to model outgoing friendship ties and triadic configurations in the network (see Appendix A4). Moreover, we estimate a model in which we use a different measure to specify the influence of the resistant behavior of friends on the adolescent’s tendency to change his/her resistance to schooling. The models are presented in Table 7. The results are highly similar to the ones we obtain in model 2 in Table 4.

**Table 7:** SIENA meta-analyses on 18 multiple group analyses on adolescents’ friendships networks and their resistance to schooling in Sweden. *N* individuals = 2480; *N* school classes = 98

	Model 1: truncated outdegree			Model 2: transitive ties			Model 3: reciprocity * transitive triplets			Model 4: average similarity		
	Coef.	S.E.	Fisher test	Coef.	S.E.	Fisher test				Coef.	S.E.	Fisher test
Network formation												
Outdegree	−2.294^****^	0.182	FL	**−2.940** ^********^	**0.640**	**FL**	−4.605^********^	0.667	FL	−4.225^********^	0.064	FL
Reciprocity	**1.540** ^********^	**0.066**	**FR**	**1.268** ^********^	**0.040**	**FR**	**1.783** ^********^	**0.083**	**FR**	**1.601** ^********^	**0.074**	**FR**
Transitive triplets	0.561^****^	0.024	FR				**0.630** ^********^	**0.028**	**FR**	**0.578** ^********^	**0.030**	**FR**
Transitive triplets * reciprocity							−0.230^********^	0.040	FL			
Transitive ties				**1.105** ^********^	**0.053**	**FR**						
3-Cycle	−0.468^****^	0.054	FL	**0.085** ^*******^	**0.038**	**FR**	−0.296^********^	0.043	FL	−0.497^********^	0.049	FL
Outdegree activity	**−0.067** ^*^	**0.029**	**FR**	**0.590** ^********^	**0.171**	**FR**	−0.094	0.177		−0.046	0.177	
Outdegree activity sqrt				**−1.181** ^**+**^	**0.653**	**FL**	1.192^+^	0.674		0.967	0.643	
Truncated outdegree (1)	−2.132^**^	0.299	FL									
Resistance alter	0.000	0.019		−0.016	0.019		−0.001	0.019		0.000	0.020	
Resistance ego	**0.024**	**0.029**		0.016	0.029		**0.021**	**0.028**		**0.021**	**0.029**	
Resistance homophily	0.408^**^	0.137	FR	0.340^*^	0.132	FR	0.354^*^	0.136	FR	0.366	0.217	FR
National-origin homophily	0.190^+a^	0.097	FR	0.206^*a^	0.088	FR	0.174^+a^	0.088	FR	0.218^*** a^	0.084	FR
Native alter	−0.140^+a^	0.072	FL	−0.128^+a^	0.071	FL	−0.137^+a^	0.071	FL	−0.147^*+* a^	0.070	FL
Native ego	0.136^a^	0.098	FR	0.129^a^	0.098	FR	0.135^a^	0.095	FR	0.116^a^	0.090	FR
Boy alter	−0.006	0.042		0.003	0.046		−0.023	0.045		−0.013^a^	0.058	
Boy ego	0.076^a^	0.096	FR	0.024^a^	0.095		0.037^a^	0.092		0.048^a^	0.092	
Sex homophily	0.426^**^	0.047	FR	0.472^**a^	0.051	FR	0.402^**a^	0.050	FR	0.430^**********a**^	0.063	FR
Behavior												
Linear shape	**−0.017**	**0.117**		−0.035	0.135		−0.029	0.135		**−0.107**	**0.138**	
Quadratic shape	**−0.069** ^*****^	**0.025**	**FL**	**−0.067** ^*******^	**0.026**	**FL**	**−0.071** ^*******^	**0.026**	**FL**	0.050^***+***^	0.027	
Resistance friends (average alt. effect)	0.244^****^	0.063	FR	**0.218** ^********^	**0.067**	**FR**	**0.227** ^********^	**0.064**	**FR**			
Resistance friends (average sim. effect)										2.685^****^	0.470	FR
Boy	**0.207** ^******^	**0.071**	**FR**	**0.225** ^********^	**0.073**	**FR**	**0.218** ^********^	**0.071**	**FR**	0.200^****^	0.067	FR
Parental education	**−0.027**	**0.025**		**−0.023**	**0.024**		**−0.026**	**0.025**		−0.033	0.025	
Status friends	−0.011	0.027		−0.007	0.031		−0.010	0.030		**0.008**	**0.031**	

Estimates and s.e.’s are obtained according to the Snijders and Baerveldt (2003) method; When the estimates and s.e.’s are printed bold, the correlation between the estimate and the s.e. is significant, and we rely on Fisher type tests. FL = P-value of Fisher’s left sided test < 0.025; FR = P-value of Fisher’s right sided test < 0.025

^a^ The estimate significantly varies across the 18 groups

^+^ *<*0.10, **<*0.05, ***<*0.01 (two-sided tests)

In the first model in Table 7, we replace the ‘outdegree activity sqrt’ effect with the ‘truncated outdegree’ effect. The truncated outdegree effect models the likelihood to nominate at least one classmate as a friend. The effect is negative, indicating that, controlled for the other effects in the model, people tend to not nominate any classmates as friends. In the questionnaire we asked people to nominate their ‘best’ friends in class. It may be that some people do not have their best friends within the class context.

In the second model we replace the transitive triplets effect with a transitive ties effect. The transitive ties effect resembles the transitive triplets effect, as it represents the tendency to befriend friends of friends (i.e., a triad is closed). However, the transitive triplets effect considers how many triads will be closed by forming a specific tie, while the transitive ties effect considers whether triads are closed by forming a specific tie.

In model 3 we add an interaction between the transitive triplets effect and the reciprocity effect (i.e., transRecTrip effect). This effect appears to be negative, indicating that the tendency of transitive closure (befriending the friends of friends) is larger for one-directional friendships.

In model 4 we measure the influence of the resistant behaviour of friends with a different effect. In the main SIENA analyses we used the average alter effect to specify this effect. This peer influence effect represents a contagion effect (Veenstra et al. 2013).^17^ In model 4 in Table 7, we use the ‘average similarity effect’ to specify the influence of the behaviour of friends. The average similarity effect indicates whether adolescents tend to minimize the difference between their own resistant behavior and the average resistant behavior of their friends (i.e., adolescents try to behave in similar ways as their friends). This effect represents a convergence type of influence. We find a positive and significant average similarity effect, indicating that adolescents try to engage in similar levels of resistance to schooling as their friends. The results obtained in model 4 in Table 7 are in line with those in model 2 in Table 4. Although the resistance homophily estimate is not significant in model 4 in Table 7, the Fisher type tests indicate that this homophily effect is positive and significant in some groups.

## Footnotes

1 Since we merely examine the increase in the gender gap in resistance to schooling in SIENA, we use a peer influence effect that is likely to contribute to the increase in the gender gap over time (see Fig. 1). In Appendix A5 we present a model in which we use a peer influence measure in SIENA that captures a convergence type of influence process.

2 We tried to estimate the influence of non-befriended classmates in SIENA. A student’s non-friends’ network in class is the mirror-image of their friendship network in class. Hence, the evolution of these networks cannot be modeled simultaneously. A SIENA model on the evolution of the non-friends network in class, assumes that the adolescent and the structure of his/her non-friends network affect who he/she does not pick as a friend. Unfortunately SIENA models on the selection of non-friends and the subsequent influence of non-friends do not converge. We considered including the resistant behavior of non-friends as a covariate in the SIENA model. However, with two waves of data, only constant covariates can be included in the model. This would assume that the network and the behavior of non-friends are static, and changes in either one of them will not be accounted for.

3 Two hundred forty six classes participated in the sociometric survey in both waves. 16 classes are dropped because the composition of the classes changed too much across the waves. An additional 121 classes are dropped, because student participation rates in either one of the two waves was too low. One class was dropped because its Jaccard index was too low.

4 Overall maximum convergence ratio <0.25.

5 In a model without interaction effects, we already estimate 7 effects in the behavioral part, and 15 effects in the friendship selection part of the model.

6 We do not include it as a time-variant characteristics, because of ceiling effects (i.e., (non-)friends with a high status may not be able to increase their status even more). Moreover, the theory does not posit that (non-)friends who increase their social status have a larger influence.

7 It is not possible to conduct a statistical test to examine whether the ‘boy’ coefficient significantly varies across the multi-level models in Stata (i.e., the suest command does not work for xtmixed models).

8 At the between-individual level in model 3, the resistant behavior of befriended and non-befriended classmates are negatively related to that of the student. These effects do not capture influence effects, but are a logical consequence of the model. Model 3 expresses the relation between the resistant behavior of friends and that of the adolescent, given the resistant behavior of non-friends. We are thus comparing students whose non-friends exhibit the same level of resistance. This makes it likely that we are comparing students who are part of the same/a similar friendship group. A student who is the most resistant person in his/her friendship group will logically have less resistant friends than a student who is the least resistant person in his/her friendship group (i.e., the relation is negative). The relation between the resistant behavior of non-friends and that of the student could be negative for similar reasons. Students who exhibit relatively high levels of resistant behavior in a class will logically have classmates who exhibit lower levels of resistant behavior than students who exhibit relatively low levels of resistant behavior in a class.

9 We also tested these interactions in separate models, this did not alter the conclusions.

10 It is not possible to conduct a statistical test to examine whether the ‘boy*time’ coefficient significantly varies across the multi-level models in Stata (i.e., the suest command does not work for xtmixed models).

11 It could be that boys increase their problem behavior in school more when friends’ resistant behavior in school is more common, while girls decrease their problem behavior more when the resistant behavior of friends is less common. If this is the case, the interaction effect will be insignificant. There are effects available in SIENA to separately model the influence of peers on an increase and a decrease in behavior. However, these effects are still under investigation for behavioral variables with more than two values, and their interpretation for such behavioral variables is still uncertain (see SIENA manual p. 37, Ripley et al. 2017).
